# Patient-specific Cardio-respiratory Motion Prediction in X-ray Angiography using LSTM Networks

**DOI:** 10.1088/1361-6560/acaba8

**Published:** 2023-01-05

**Authors:** Fariba Azizmohammadi, Iñaki Navarro Castellanos, Joaquim Miró, Paul Segars, Ehsan Samei, Luc Duong

**Affiliations:** 1 Interventional Imaging Lab, Department of software and IT engineering, École de Technologie Supérieure, 1100 Notre-Dame Street West, Montreal, Quebec, Canada H3C 1K3, Canada; 2 Department of Pediatrics, CHU Sainte-Justine, Montreal, Canada H3T 1C5, Canada; 3 Department of Radiology, Carl E. Ravin Advanced Imaging Laboratories, Duke University Medical Center, Durham, NC, United States of America

**Keywords:** cardiac motion, respiratory motion, cardio-respiratory motion prediction, X-ray angiography, LSTM model, motion tracking

## Abstract

**Objective.:**

To develop a novel patient-specific cardio-respiratory motion prediction approach for X-ray angiography time series based on a simple long short-term memory (LSTM) model.

**Approach.:**

The cardio-respiratory motion behavior in an X-ray image sequence was represented as a sequence of 2D affine transformation matrices, which provide the displacement information of contrasted moving objects (arteries and medical devices) in a sequence. The displacement information includes translation, rotation, shearing, and scaling in 2D. A many-to-many LSTM model was developed to predict 2D transformation parameters in matrix form for future frames based on previously generated images. The method was developed with 64 simulated phantom datasets (pediatric and adult patients) using a realistic cardio-respiratory motion simulator (XCAT) and was validated using 10 different patient X-ray angiography sequences.

**Main results.:**

Using this method we achieved less than 1 mm prediction error for complex cardio-respiratory motion prediction. The following mean prediction error values were recorded over all the simulated sequences: 0.39 mm (for both motions), 0.33 mm (for only cardiac motion), and 0.47 mm (for only respiratory motion). The mean prediction error for the patient dataset was 0.58 mm.

**Significance.:**

This study paves the road for a patient-specific cardio-respiratory motion prediction model, which might improve navigation guidance during cardiac interventions.

## 1. Introduction

Cardiovascular disease (CVD) is a leading cause of death worldwide today (Scott *et al* 2013). Image-guided interventional procedures are minimally invasive treatments for CVD and have been gaining popularity in the last two decades. Catheter-based procedures using fluoroscopy are prevalent within image-guided interventions and are becoming increasingly complex modern tools for planning and guiding cardiac interventions (Ackermann and Ender 2019). Fluoroscopy is commonly used in the navigation of cardiac interventions. In the process, 2D X-ray images are acquired in real-time during the visualization of contrasted arteries. It provides adequate temporal and spatial information from the visible targets in images (contrasted arteries, medical devices, etc) (Dauer 2011).

During X-ray acquisitions, multiple organs, including the arteries, move with the heartbeat, respiration and with occasional unexpected patient movements. Such movements cause artifacts in the image acquisition process and render the navigation process challenging. Overall, image-guided cardiac interventions are greatly affected by cardio-respiratory motion (Shechter *et al* 2004, McClelland *et al* 2013). The cardiac motion, being distinct for each patient, and hardly perceptible on angiographies, generates additional complexity for cardiologists. Moreover, respiratory motion degrades the detection and quantification capabilities of imaging modalities. Mismatches between some imaging techniques due to respiratory motion also result in additional attenuation correction (AC) artifacts and inaccurate localization.

Accurate image-guided treatments in CVDs are very important. Thus, an accurate method ensuring the reduction of the effects of respiratory, cardiac, and unexpected motions during cardiology interventions is needed. In recent years, numerous methods have been proposed for controlling respiratory and cardiac motion, as well as for minimizing its effects.

One approach that has been put forward to overcome the problems caused by organs moving during image acquisitions and image-guided interventions is called breath-holding. With this, the respiratory motion is suppressed by having a patient to practice multiple short breath-holds. However, when a breath-hold ends, the patients chest and internal organs move due to realignment. There are other limitations, such as scan time constraints in data acquisition that can limit the image signal-to-noise ratio and accordingly spatial resolution and poor steady breath-holding with this technique (Oshinski *et al* 1996). Given these limitations of breath-holding techniques, free-breathing techniques are more popular these days. Thus, there has been more focus on improving free-breathing methods (Taylor *et al* 1997, Nehrke *et al* 2001).

Other methods propose solutions to estimate and predict cardio-respiratory motion during image acquisition. Image-based cardio-respiratory motion estimation approaches are mainly based on surrogate data tracking. Diaphragm tracking for respiratory motion and catheter tracking for cardiac movements are the most popular image-based motion estimation approaches aimed at making up for the effects of cardio-respiratory motion during image-guided interventions (Schweikard *et al* 2000, Shechter *et al* 2005, Timinger *et al* 2005, Kesner and Howe 2010, Ma *et al* 2011). However, these approaches usually require multi-modality imaging (ultrasonic, X-ray fluoroscopy, and magnetic resonance imaging (MRI)) (Roujol *et al* 2013, Baka *et al* 2015). Several methods have been developed to segment and track visible medical devices under X-ray images in realtime. Deep convolutional neural networks are applied in approaches performing realtime tracking of the segmented catheters and guidewires in 2D X-ray fluoroscopic sequences. For input, the network takes the current and the three previous images and segments the catheter and guidewire in the current image (Ambrosini *et al* 2017).

Motion prediction strategies can be either model-based, model-free, or hybrid. Model-based motion prediction algorithms propose a mathematical model such as linear prediction, Bayesian filtering, sinusoidal model, finite state model, etc. A motion model takes surrogate data as input and estimates the motion as output (Gierga *et al* 2005, Ernst and Schweikard 2009, Werner *et al* 2009, Riaz *et al* 2009, Ruan and Keall 2010, Kalet *et al* 2010, Schneider *et al* 2010, Jung *et al* 2013). Model-free methods are learning-based methods that use a large amount of data and artificial neural network structures to find a pattern for cardio-respiratory motion that predict the motion signal (Sharp *et al* 2004, Dieterich and Murphy 2006, Pokhrel and Murphy 2009). For the respiratory motion prediction, there are learning-based methods presented in (Lee and Motai 2014) that have achieved less than a 2mm prediction error, which is promising for cardiac applications. Hybrid motion prediction algorithms combine model-based and model-free methods and leverage the advantages of both algorithms, which is why they can outperform the individual methods (Feldkamp and Puskorius 1994, Respiratory motion prediction by using the adaptive neuro fuzzy inference system (ANFIS), 2005, Schilling *et al* 2007).

## 2. Proposed contribution

We present a novel patient-specific cardio-respiratory motion prediction approach using a simple supervised long short-term memory (LSTM) network for angiography sequences. The 2D displacements of the moving objects in an angiography sequence are extracted from the images and represented through 2D affine transformation matrices. Then, a many-to-many LSTM model is trained for every sequence to predict the next geometrical transformation of the arteries in the future frames of the sequence from previous ones.

## 3. Materials and methods

2D transformation parameters (translation, rotation, scaling, and shearing) are extracted from the X-ray angiography sequences representing the motion features in matrix form. Thus, a sequence of affine transformation matrices resulting from frame-by-frame image registrations of the original X-ray sequence is fed into the sequential LSTM model for training. Then, the future displacements of the moving targets (contrasted arteries and medical devices) in the upcoming frames are predicted.

### 3.1. Data description

This approach was developed with a simulated X-ray angiography dataset using a realistic XCAT computational phantom simulator (Segars *et al* 2010, 2013) and was validated with real patient X-ray angiography sequences from Sainte-Justine Hospital.

Computerized phantoms play a major role in medical imaging research today. They are very helpful in that they provide a practical means for evaluating and improving imaging techniques and devices. In this study, we employed realistic XCAT computational phantoms with the cardio-respiratory motion for both adult and pediatric patients. Initially, our experiments were done on three different adult patients (Azizmohammadi *et al* 2019). The length of the sequences used to capture at least two to five heart and/or respiratory cycles varied between 75 and 150 frames. For each patient, three different types of motions (only cardiac, only respiratory, and both motions) were generated. Respiratory motion is not gated with cardiac motion, and the misalignment between the motions makes the motion estimation more complicated. Therefore, in this method, we simulated different motion modes to assess the predictions based on the motion complexity. Simulations were also carried out for different circumstances, with the patient having normal and abnormal respiratory and heartbeat cycles. A normal heartbeat cycle is 1 a second long, while the respiratory cycle, is 5 seconds. These values can vary between patients and change if the patient is under stress or not breathing normally. We then simulated 64 pediatric and adult patients (36 male and 28 female) falling within the 8-month newborn to 85-year age range, including the left and right coronary arteries. The pediatric simulated dataset included 112 sequences (2 sequences per patient, showing either the left coronary artery or right coronary artery), while our adult simulated dataset included 12 sequences.

For the real patient dataset, we selected 10 different sequences with lengths ranging between 84 and 166 frames and having visible moving objects (contrasted arteries and/or medical devices such as catheters and guide wires).

### 3.2. Preprocessing: segmentation and centerline extraction of simulated and patient X-rays sequences

To track the motion signal in an X-ray sequence, pre-processing steps were applied to segment and extract the centerlines of the moving targets. Using the Frangi filter and the connected components, we segmented the arteries and skeletonized the segmentation to extract the centerlines. The 2D motion features were extracted by registering the X-ray images, frame-by-frame in the sequence.

**Figure 1.**
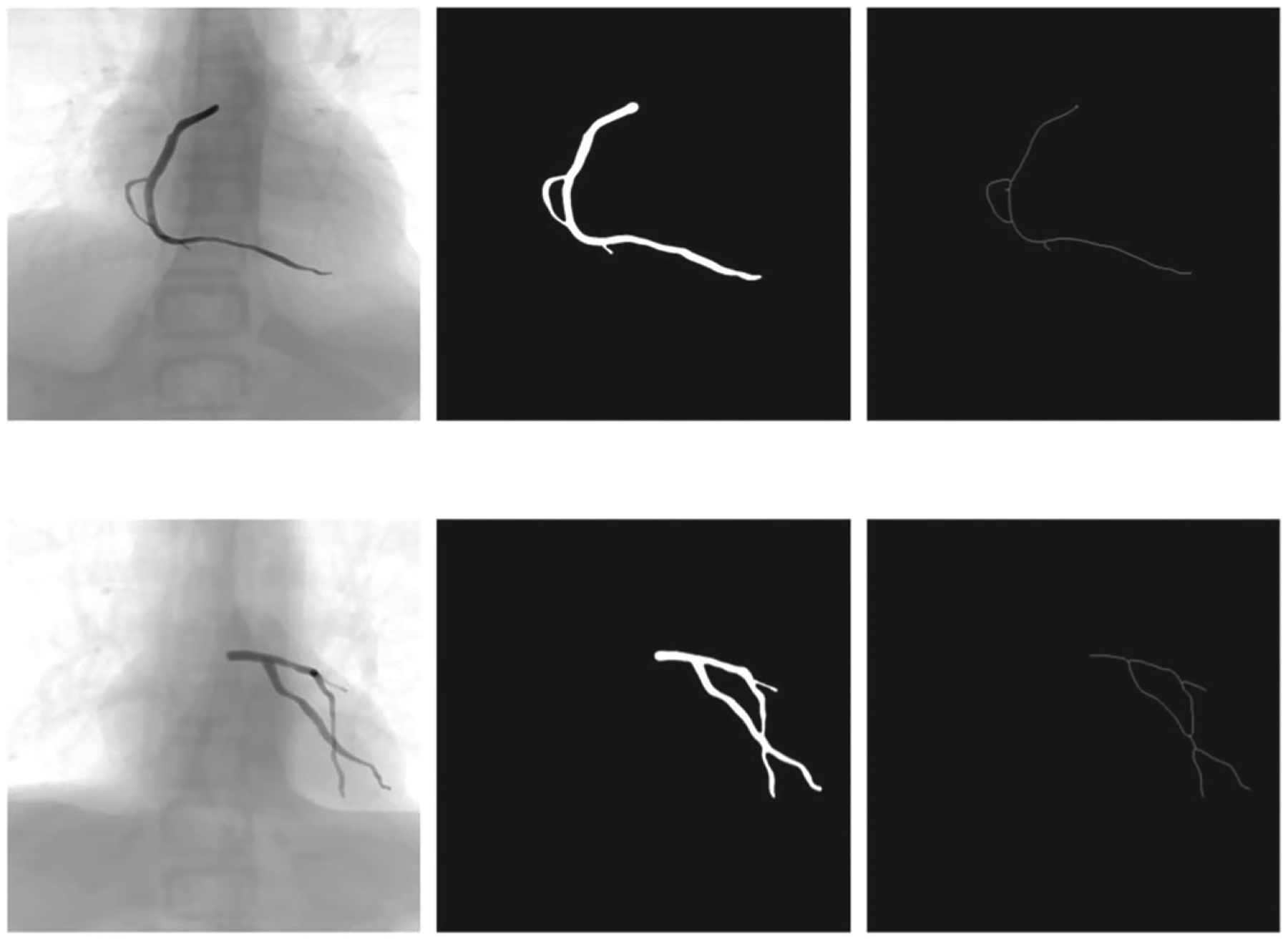
Pre-processing steps (segmentation, denoising, and centerline extraction) on simulated data. The first row is a Right Coronary Artery (RCA) branch and the second row shows the Left Coronary Artery (LCA).

**Figure 2.**
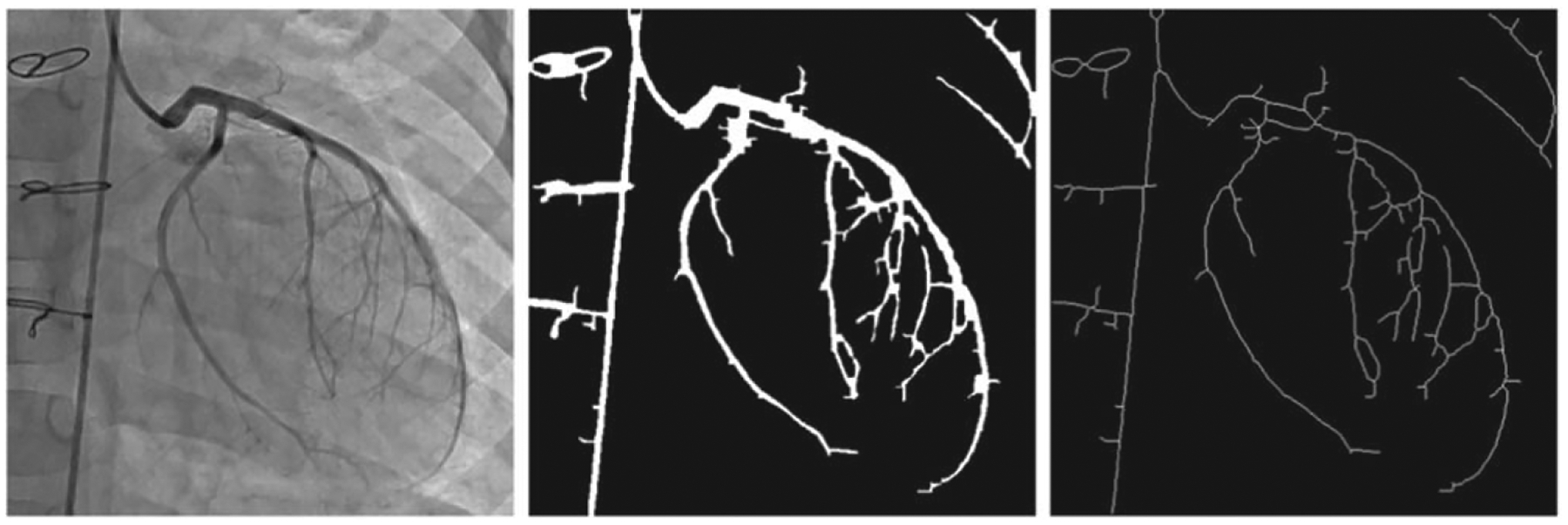
Pre-processing steps on patient data (segmentation, denoising, and centerline extraction).

For the simulated data, the vascular structures were extracted from the image frames for each sequence and segmented by applying image processing filters and connected components to remove the small objects. Figures 1 and 2 show the vessel structures extracted from the original X-rays (simulated and patient data, respectively). The Frangi filter parameters were found experimentally. For the scale parameter (sigma), an interval of [1, 6] was considered with a step size of 0.1. These values for the parameters were set based on the visual inspection of the images in both simulated and patient datasets.

We applied the same segmentation steps for the patient data, although the segmentation was noisy in the background as compared to the simulated images. Moreover, since the arteries’ structures were not continuously contrasted in all the image frames of a given sequence, we segmented medical devices as moving objects, where the arteries were not visible or faded in some frames. We changed the Frangi filter parameters as a function of the visible objects in the patient sequences.

#### 3.2.1. Coherent point drift (CPD) registration

The point set registration algorithm is widely used in computer vision problems such as image registration. The registration can be rigid or non-rigid. Given two point sets (centerlines of two consequent frames), we applied CPD as a non-rigid registration to map one point set to the other, yielding a non-rigid transformation. Non-rigid transformations include affine transformations such as scaling and shear, as well as translation and rotation mapping.

The CPD algorithm is a Gaussian Mixture Model (GMM) based algorithm that assigns correspondence points between two sets of point clouds. It retrieves the transformations for mapping each point cloud to the other using a specified registration (Myronenko and Song 2010). The alignment of the two point clouds is a probability density estimation problem. The first point set is centered on the second one by fitting the GMM algorithm and maximizing the likelihood. The GMM moves coherently and retains the topological structure of the point sets (Myronenko and Song 2010). A coherence constraint is imposed for affine registration by re-parametrizing the GMM centroid locations with affine transformation parameters (translation, rotation, shearing, scaling). These parameters are concatenated to build the Affine Transformation (AT) matrix as follows:

(1) $$AT=\left[\begin{array}{ccc} s_{x}\text{ cos}(\theta ) & s_{y}\text{ sin}(\theta ) & x-c_{x}s_{x}\text{ cos}(\theta )-c_{y}s_{y}\text{ sin}(\theta )\\ -s_{x}\text{ sin}(\theta ) & s_{y}\text{ cos}(\theta ) & y+c_{x}s_{x}\text{ sin}(\theta )-c_{y}s_{y}\text{ cos}(\theta )\\ 0 & 0 & 1 \end{array}\right]$$

(2) $$ATM=\left[\begin{array}{ccc} A00 & A01 & Tx\\ A10 & A11 & Ty\\ 0 & 0 & 1 \end{array}\right].$$

While *A*_00_ = *s*_*x*_ cos(*θ*), *A*_01_ = *s*_*y*_ sin(*θ*), *A*_10_ = −*s*_*x*_ sin(*θ*), *A*_11_ = *s*_*y*_ cos(*θ*), *T*_*x*_ = *x* − *c*_*x*_*s*_*x*_ cos(*θ*) − *c*_*y*_*s*_*y*_ sin(*θ*) and *T*_*y*_ = *y + c*_*x*_*s*_*x*_ sin(*θ*) − *c*_*y*_*s*_*y*_ cos(*θ*). We used notations *A*_00_, *A*_01_, *A*_10_, *A*_11_, *T*_*x*_, *T*_*y*_ for the predicted parameters. The extracted centerlines of the arteries are considered as bright point sets. Every centerline point set in each frame is registered to the previous frame in a sequence using the CPD algorithm.

### 3.3. LSTM models for sequence prediction

LSTM is a recurrent neural network (RNN)-based architecture with optimized memory which can solve the vanishing and exploding gradient problem in conventional RNNs. LSTM structures have memory blocks which include memory cells that can store the temporal information of sequential data as well as specific multiplicative units, called gates, to control the flow of information. Each memory block contains an input gate to control the flow of input activations into the memory cell, an output gate to control the output flow of cell activations into the rest of the network, and a forgetting gate. Therefore, a LSTM network can keep only the necessary information from the past and forget the rest, thus optimizing its memory.

**Figure 3.**
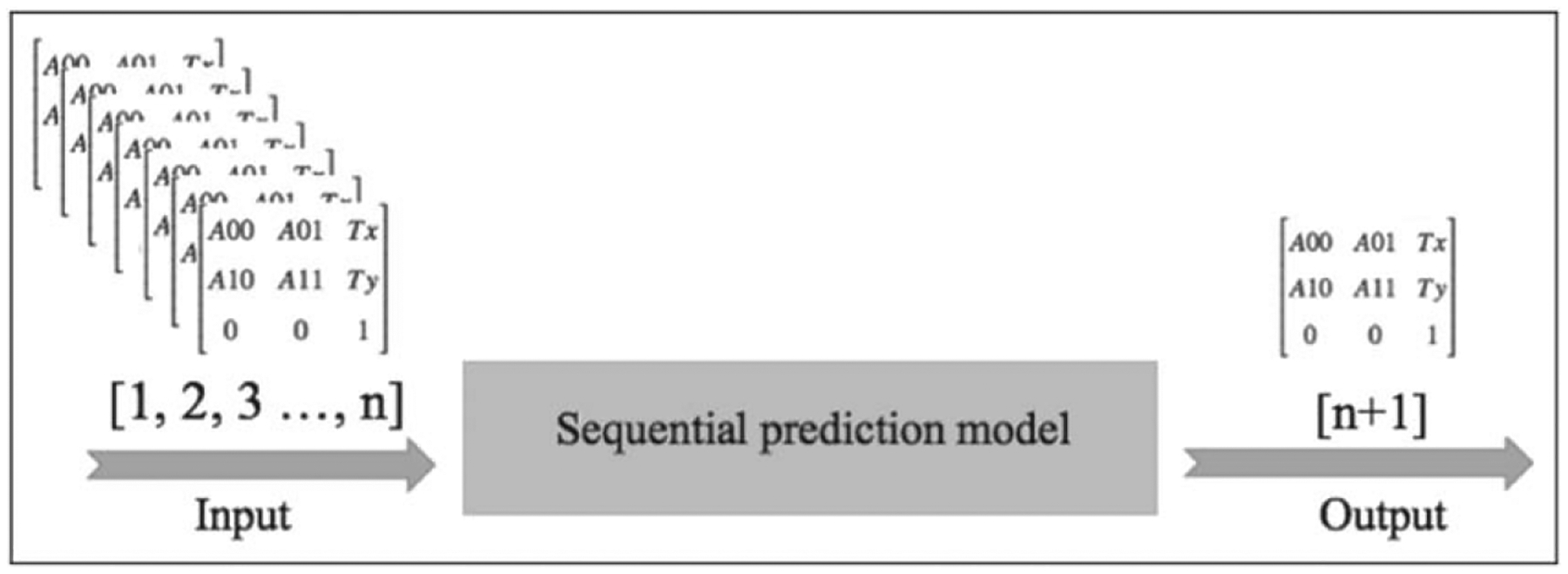
Many-to-one structure of the sequential prediction.

**Figure 4.**
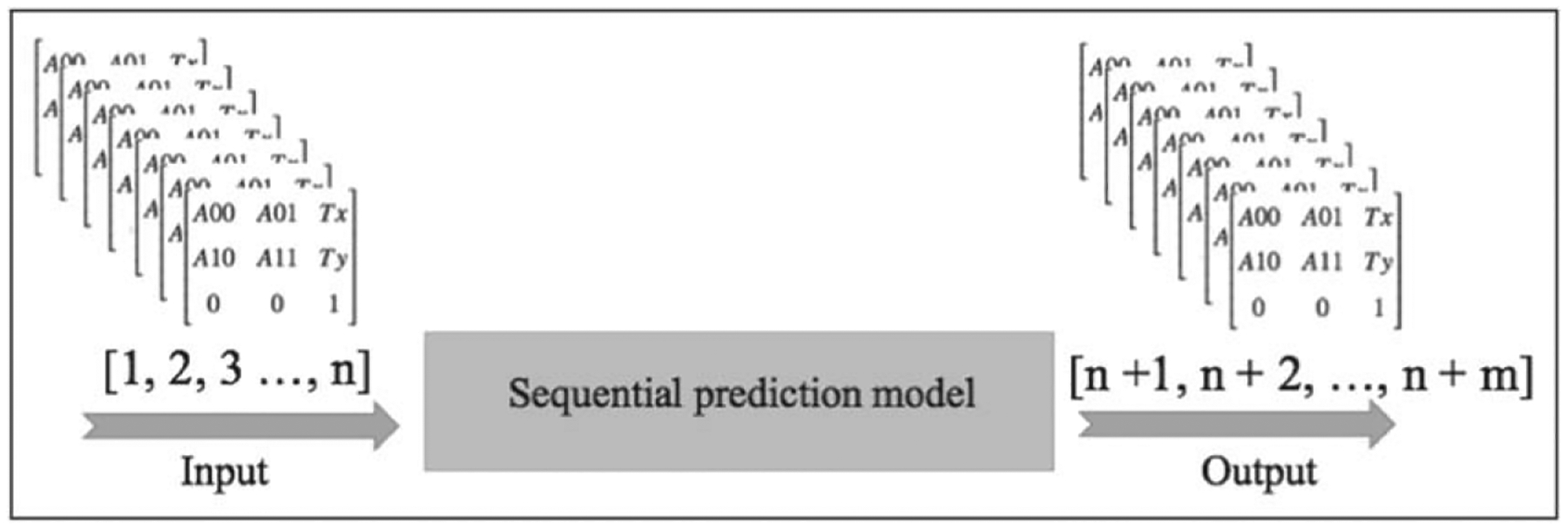
Many-to-many structure of the sequential prediction.

Initially, the model could predict the transformation matrix for a single future frame given the previous frames using a many-to-one LSTM structure such that given a sequence of frames as an input, we were expecting one single frame as output. The new proposed model can forecast multiple values in the future after receiving multiple inputs to improve time complexity. Figures 3 and 4 show the structures of many-to-one and many-to-many frame prediction respectively. In both models, the input for the model is a sequence of transformation matrices extracted from the X-ray images by registering the consequent frames in pairs, and the LSTM network is trained to predict the arteries’ transformation in the next frame/s from the previous ones.

Considering *N* = 6 as the number of 2D affine transformation parameters representing translation, rotation, shearing, and scaling (*Tx*, *Ty*, *A*00, *A*01, *A*10, *A*11), and *T* as the number of transformation matrices. To effectively feed the models we sort the parameters in a vector *X*^*t*^ of size N*T. This vector is called the transformation parameter vector (TP). Then, the values in the TP vector are normalized to be fed into the network. The normalization was required since the range of values for some parameters is so small or big and in that case, the network can not learn or converges slowly. Then, at the end of the prediction, they can be de-normalized to have the actual values. Now, the prediction problem is defined as solving the predictor of *X*^*t*^ (denoted by ${\hat{X}}^{t}$) via a series of previously measured TP vectors.

**Figure 5.**
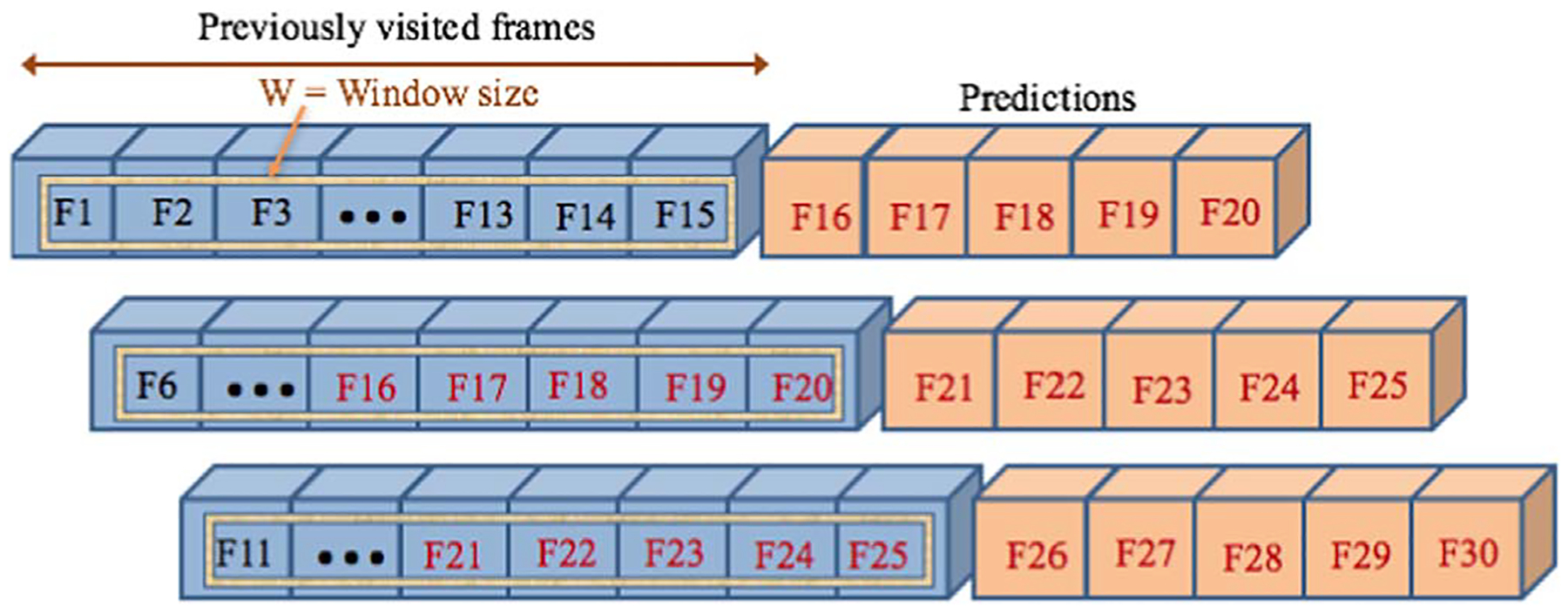
The many-to-many LSTM model. All TP elements are predicted at the same time. *W* = 15 is the window size and the number of outputs or predictions is *P* = 5.

Compared to the primarily (many-to-one) model, the new (many-to-many) model was developed to predict all the *N* = 6 transformation parameters at the same time, for multiple matrices. In the primarily (many-to-one) model, we assumed that all the parameters were independent of each other. Thus, to predict the vector *X*^*t*^, the model was able to predict only one element or parameter ${\text{x}}_{n}^{t}$ at a time by feeding the LSTM one vector $\left(x_{0}^{t},x_{1}^{t},\ldots ,x_{n}^{t}\right)$ of size *n* = *T* at a time. Yet, in the new model, the many-to-many model could predict all *N* = 6 parameters simultaneously. To feed the input matrix sequences to the model, a fixed number of previous frames (matrices) including enough information (at least one heart cycle) was considered as a time window from which to learn to predict the new TPs in the future (figures 5).

**Figure 6.**
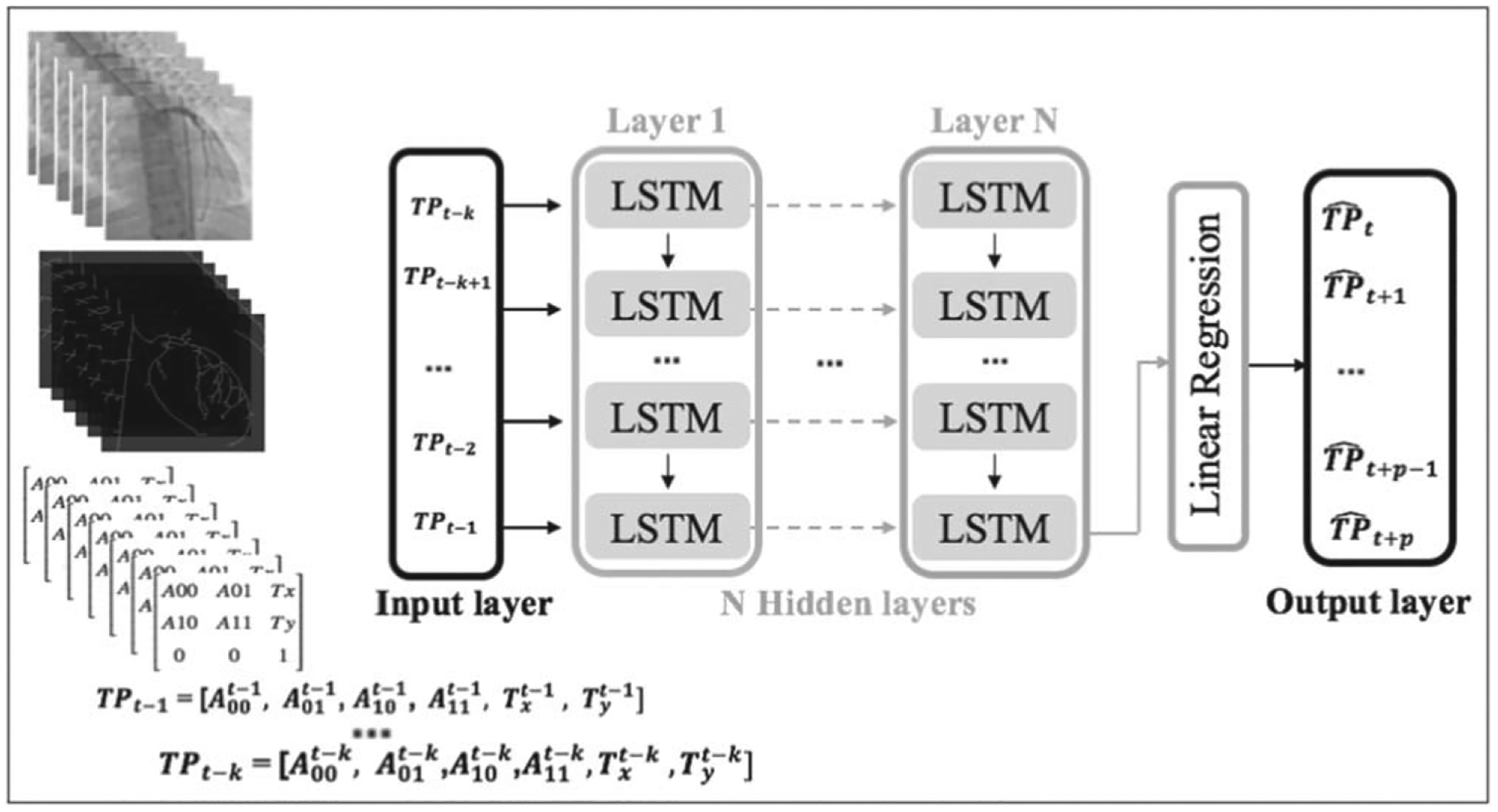
The deep LSTM model structure for multiple TP predictions. The required pre-processing steps to extract the input matrics are shown on the left. The metrics are flattened to be fed into the input layer. The network has N = 500 hidden layers and a linear regression layer to generate multiple outputs.

Figure 6 shows the deep LSTM model structure. First, the input image sequence is pre-processed and the TP vectors are extracted and then the deep LSTM model is fed by multiple TP vectors. The output of the N-layer LSTM model is passed by a linear regression layer and the output of the model is multiple future TP vectors.

## 4. Experiments and results

We used 64 patients (pediatric and adult) simulated in normal and abnormal modes for different motions (cardiac only, respiratory only, and both motions). The sequences simulated with both motions were 75 frames in length, while other sequences having only cardiac or only respiratory motion were generated using a 150 frame length. Additionally, 10 sequences from the patient dataset with between 84 and 166 frames in length were selected based on the visibility of contrasted arteries or medical devices through the sequences.

The motion features extracted from the centerlines of the segmented arteries were represented as 6 affine transformation parameters collectively called the TP vector translations Tx, Ty, and rotation, shearing, scaling was considered as 15 matrics to capture at least two heart cycles and one breathing cycle in average. The number of predictions with the model was set to 5 (one over third of the window size). For each sequence, the LSTM model was trained separately with a sequence of TP vectors, while 80 percent of each sequence was considered as the training set and 20 percent as the testing set. TP vectors were normalized by dividing by the maximum value in each TP vector. Since the prediction is considered a regression problem, we used a linear activation function for our model and the RMSProp as an optimizer for compilation. Based on the experiments, the optimal number of epochs within the 100 to 1000 range was 500 for the simulated data with a length of 150 frames and 200 for sequences with 75 frames. We trained the patient data sets with 500 epochs as well.

**Figure 7.**
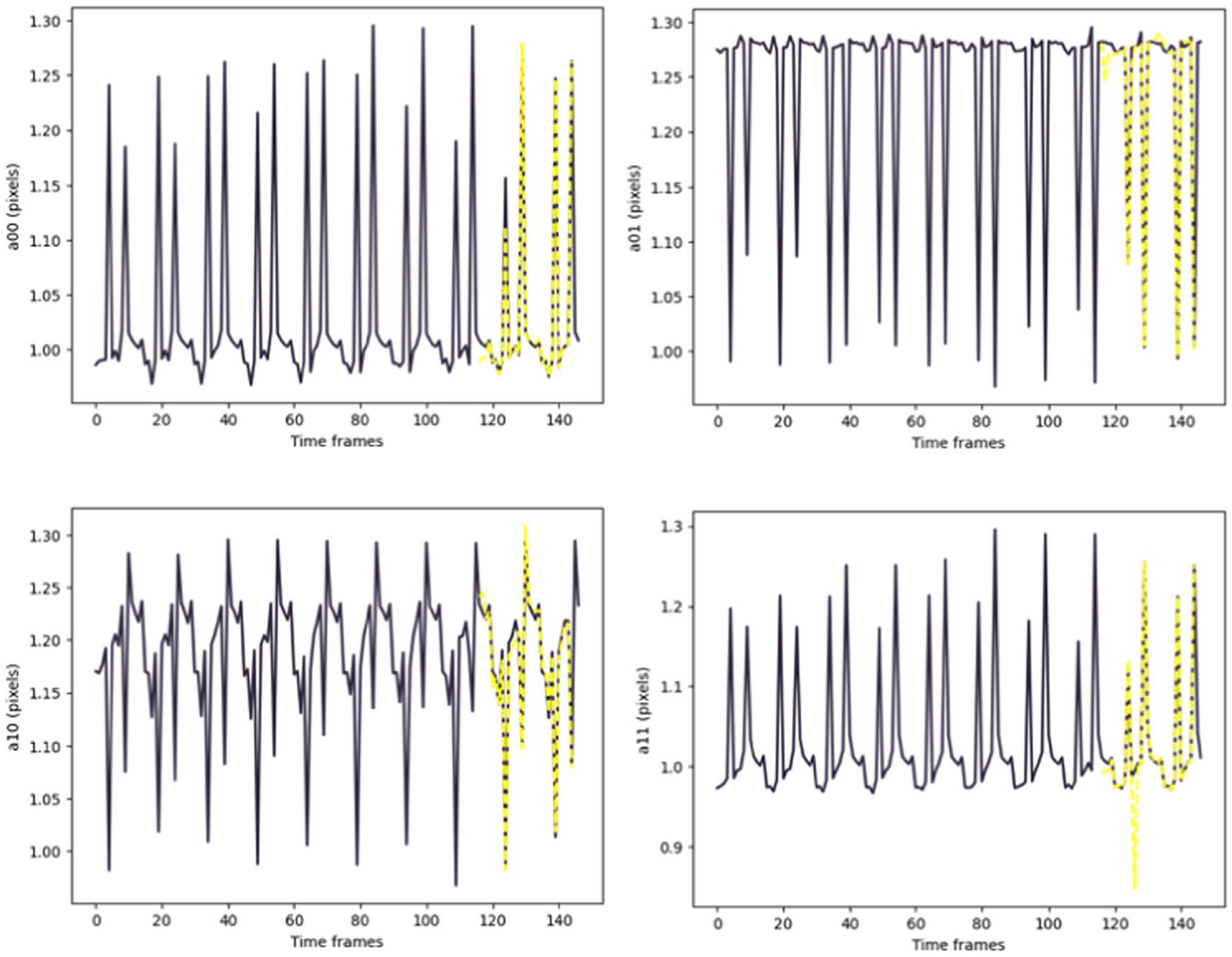
Simulated data example for prediction of 2D transformation parameters (*A*00, *A*01, *A*10, *A*11) for moving arteries with both cardiac and respiratory motions. The ground truth values are shown in dark purple, while the yellow dashed lines show the predictions.

**Figure 8.**
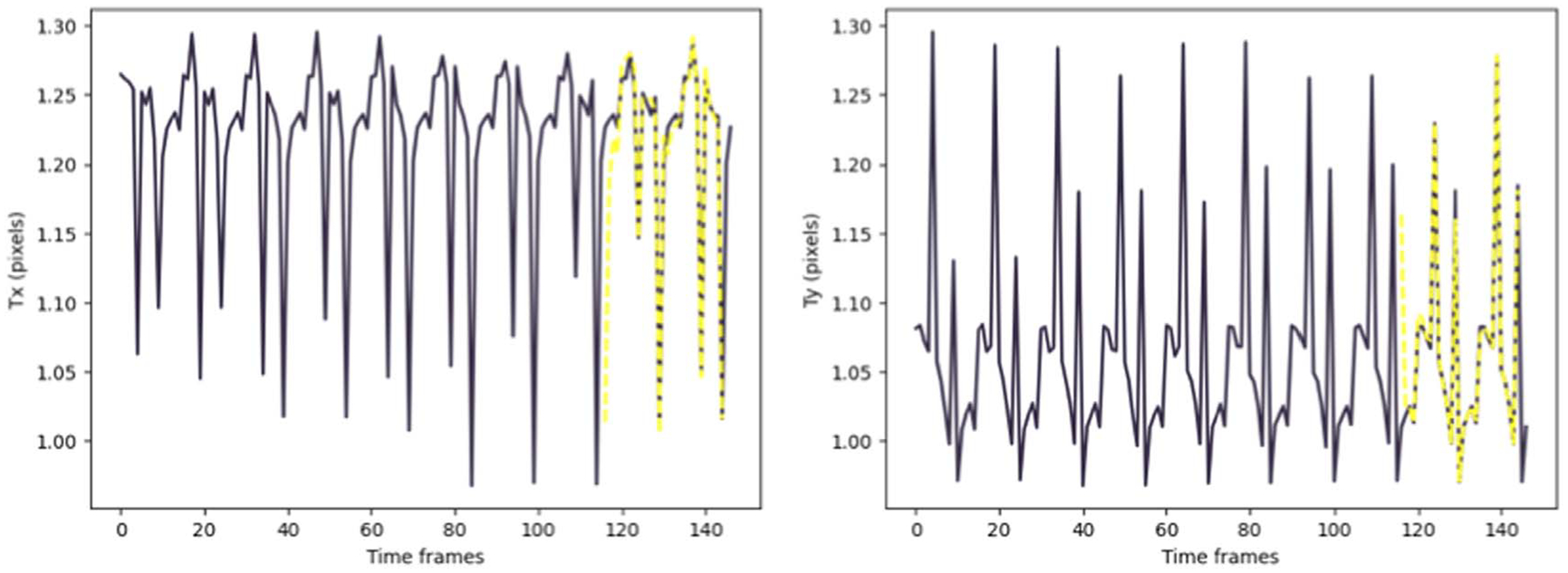
Simulated data example for prediction of 2D translation parameters (*Tx*, *Ty*) for moving arteries with both cardiac and respiratory motions. The ground truth values are shown in dark purple, while the yellow dashed lines show the predictions.

**Figure 9.**
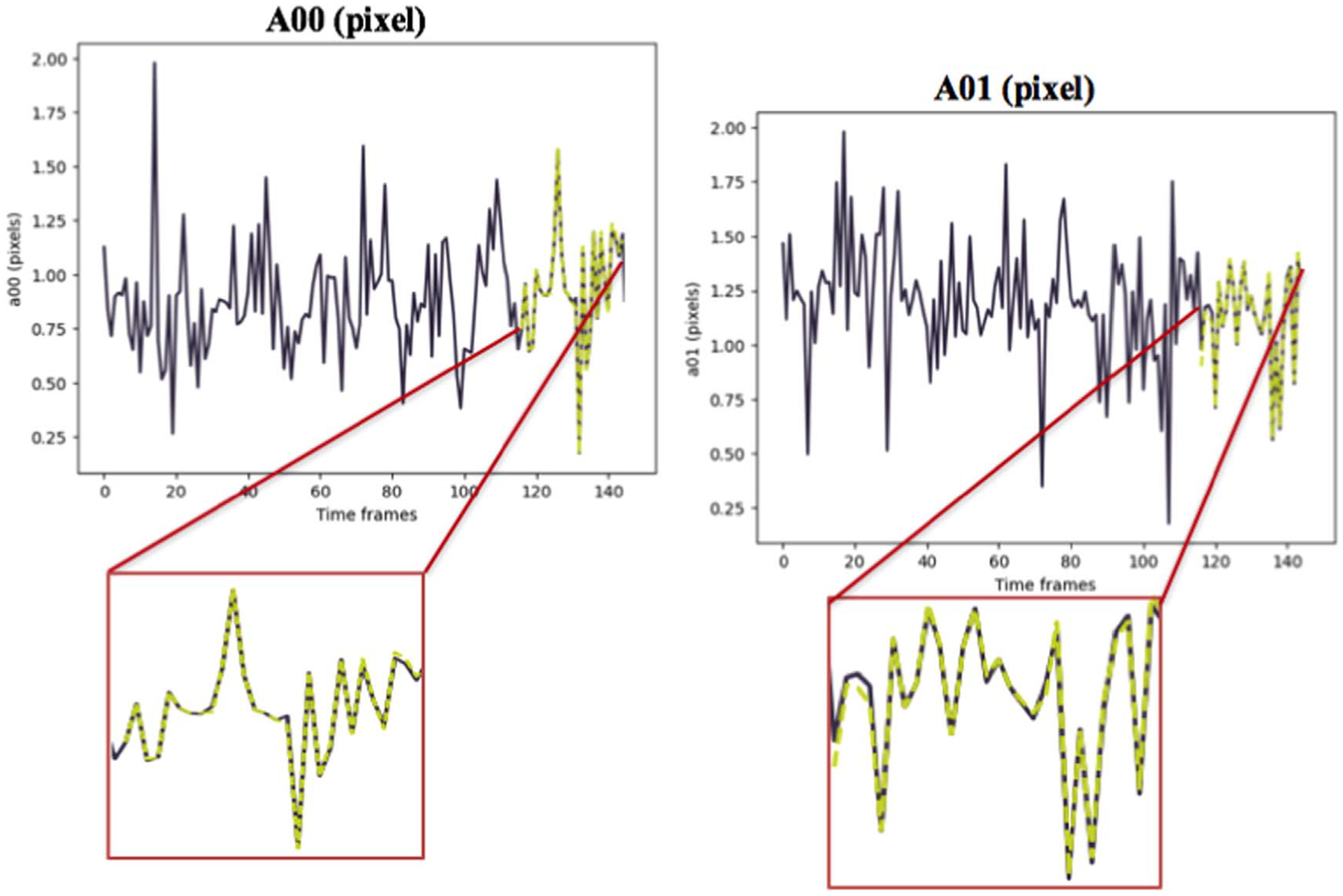
Patient data example for prediction of 2D transformation parameters (*A*00, *A*01) for moving arteries with both cardiac and respiratory motions. The ground truth values are shown in dark purple, while the yellow dashed lines show the predictions.

**Figure 10.**
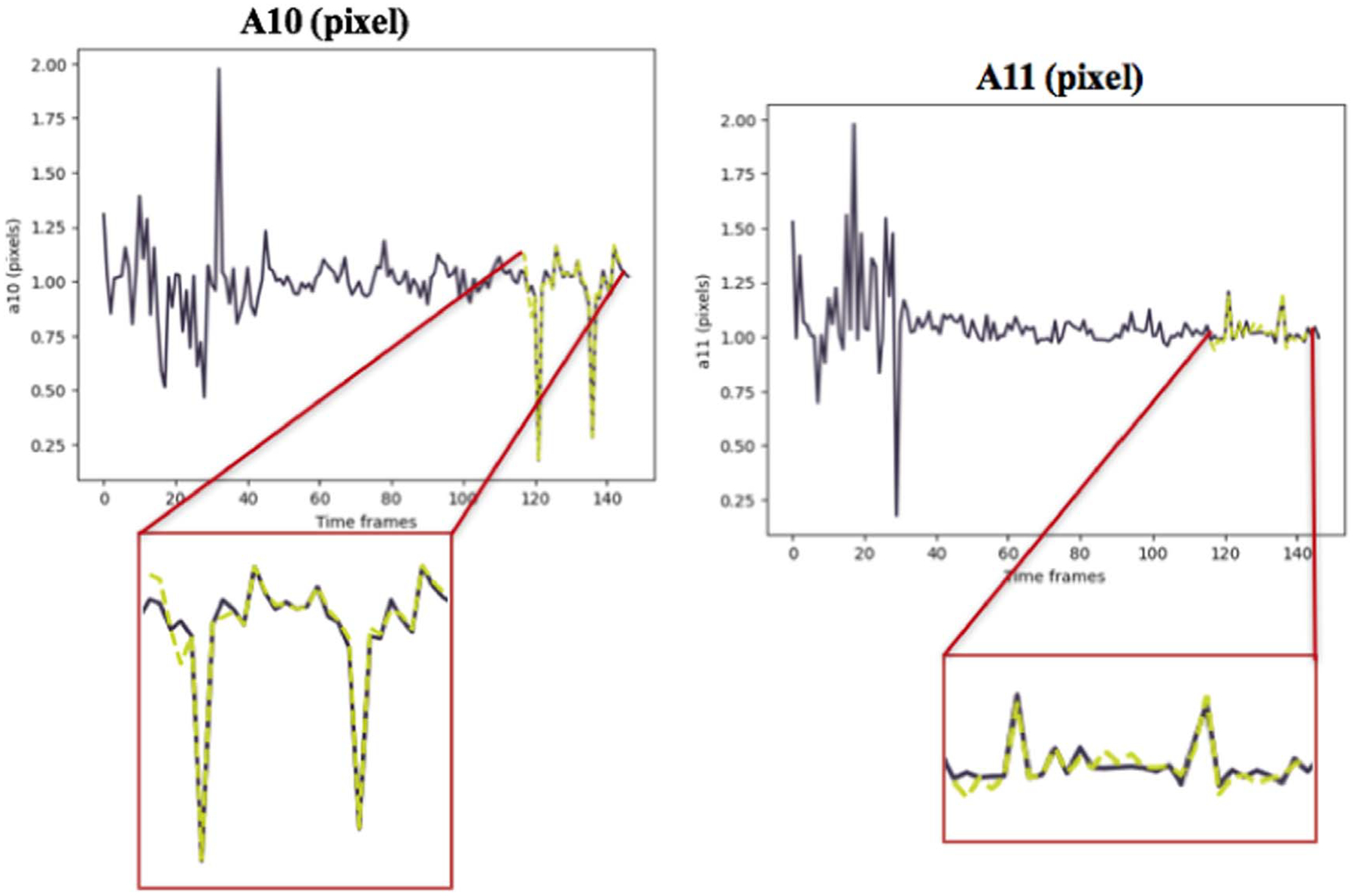
Patient data example for prediction of 2D transformation parameters (*A*10, *A*11) for moving arteries with both cardiac and respiratory motions. The ground truth values are shown in dark purple, while the yellow dashed lines show the predictions.

**Figure 11.**
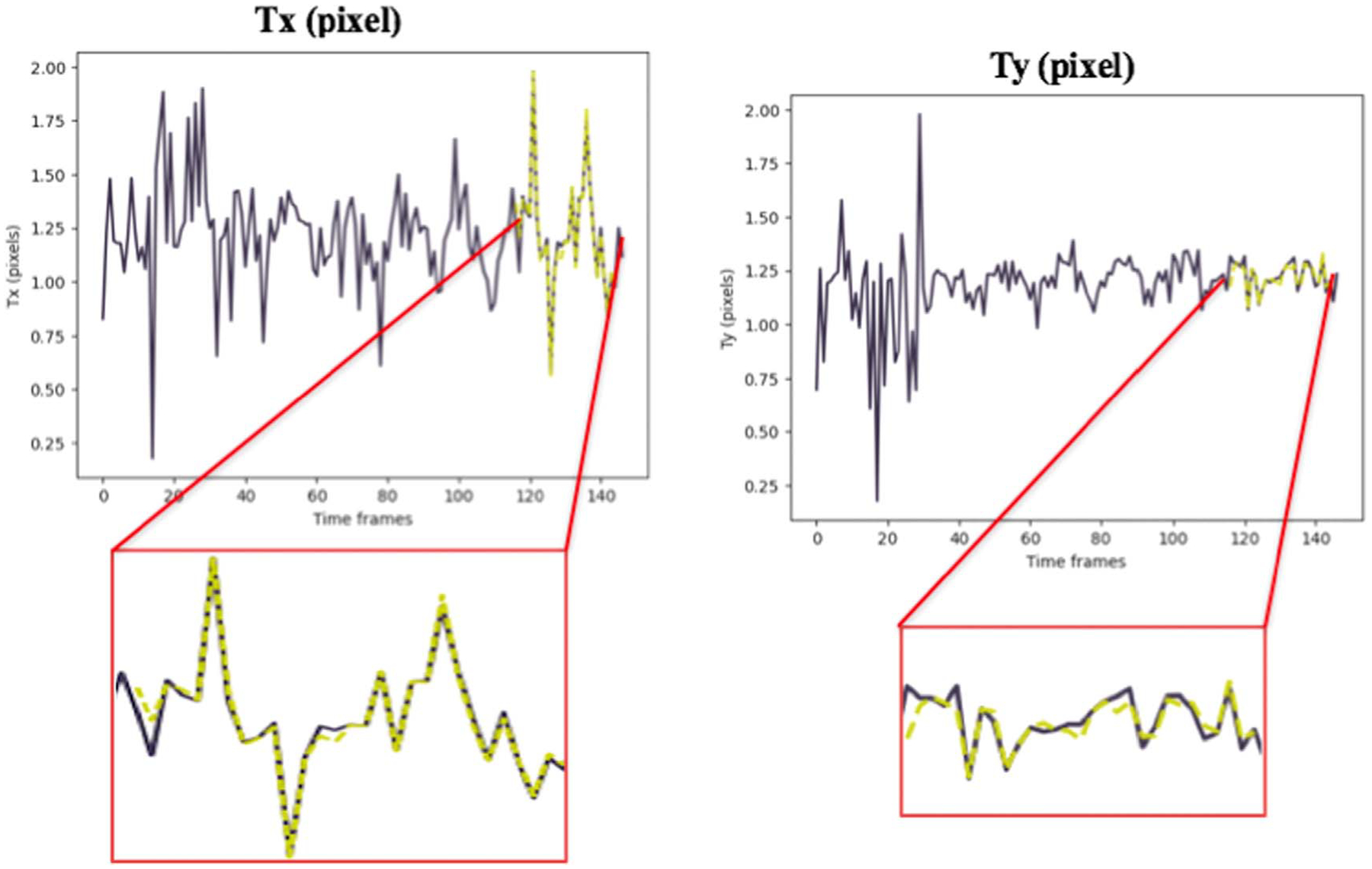
Patient data example for prediction of 2D translation parameters (*Tx*, *Ty*) for moving arteries with both cardiac and respiratory motions. The ground truth values are shown in dark purple, while the yellow dashed lines show the predictions.

Keras library was used to build and train the model. The accuracy of the method was evaluated by computing the Mean Absolute Error (MAE) between the predicted values using our model and the results of the CPD registration as the ground truth (tables 1 and 2). Figures 7 and 8 depict the TP predictions for a simulated data sample and figures 9, 10 and 11 show a patient data TP prediction sample. The patient dataset contains motion irregularities, as illustrated in figures 10 and 11. Nevertheless, the imposed patient movement represented as irregularities in the motion signal did not affect the predictions.

To evaluate the overall error of predicted transformed centerlines, we first calculated the distance transform of the original centerline images. For each pixel of the background, we obtained its distance to the closest centerline point. The distance transform or distance field for each white pixel on the extracted centerline assigns a number, which is the distance between that pixel and the nearest nonzero pixel of the vessels. Thus, to calculate the final distance, we projected the predicted transformed centerline on the distance transform matrix and averaged the obtained values as the overall prediction error.

**Figure 12.**
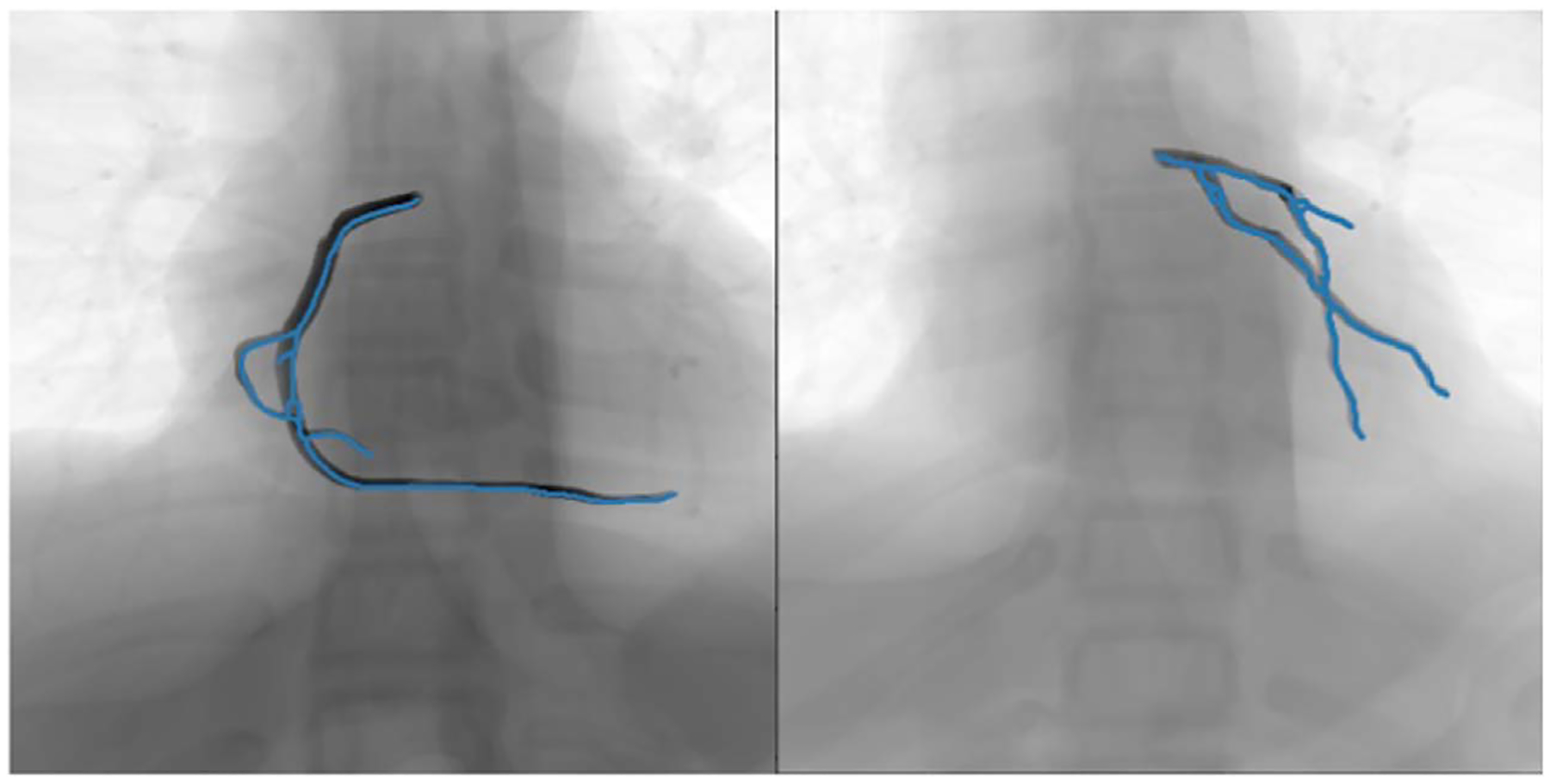
Simulated data example for overlaying the transformed LCA and RCA arteries with predicted transformation parameters (blue colored centerlines) with the original transformed images.

**Figure 13.**
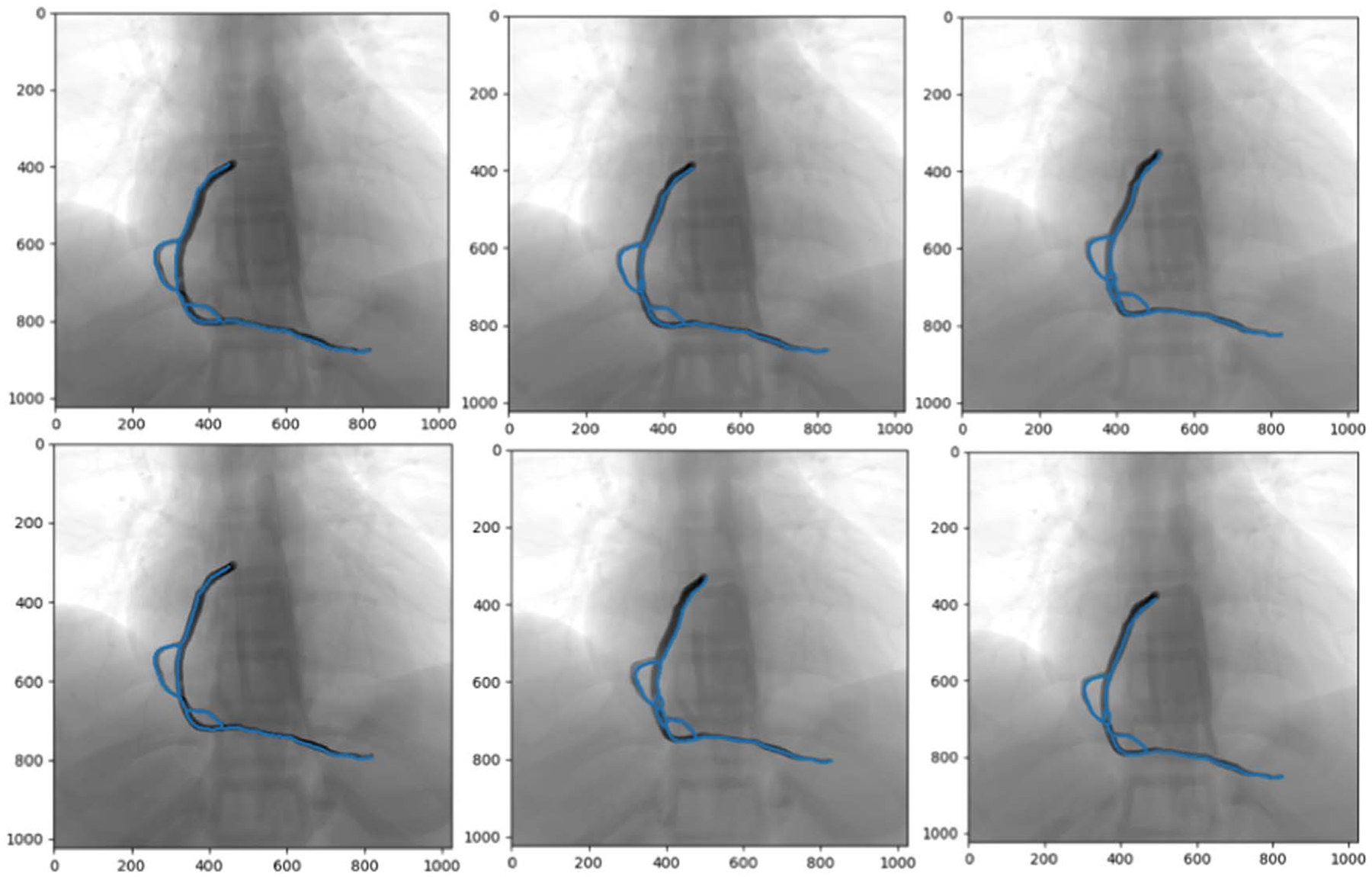
Simulated data example for overlaying the transformed arteries in 6 consecutive frames with predicted transformation parameters (blue colored centerlines) with the original transformed images.

**Figure 14.**
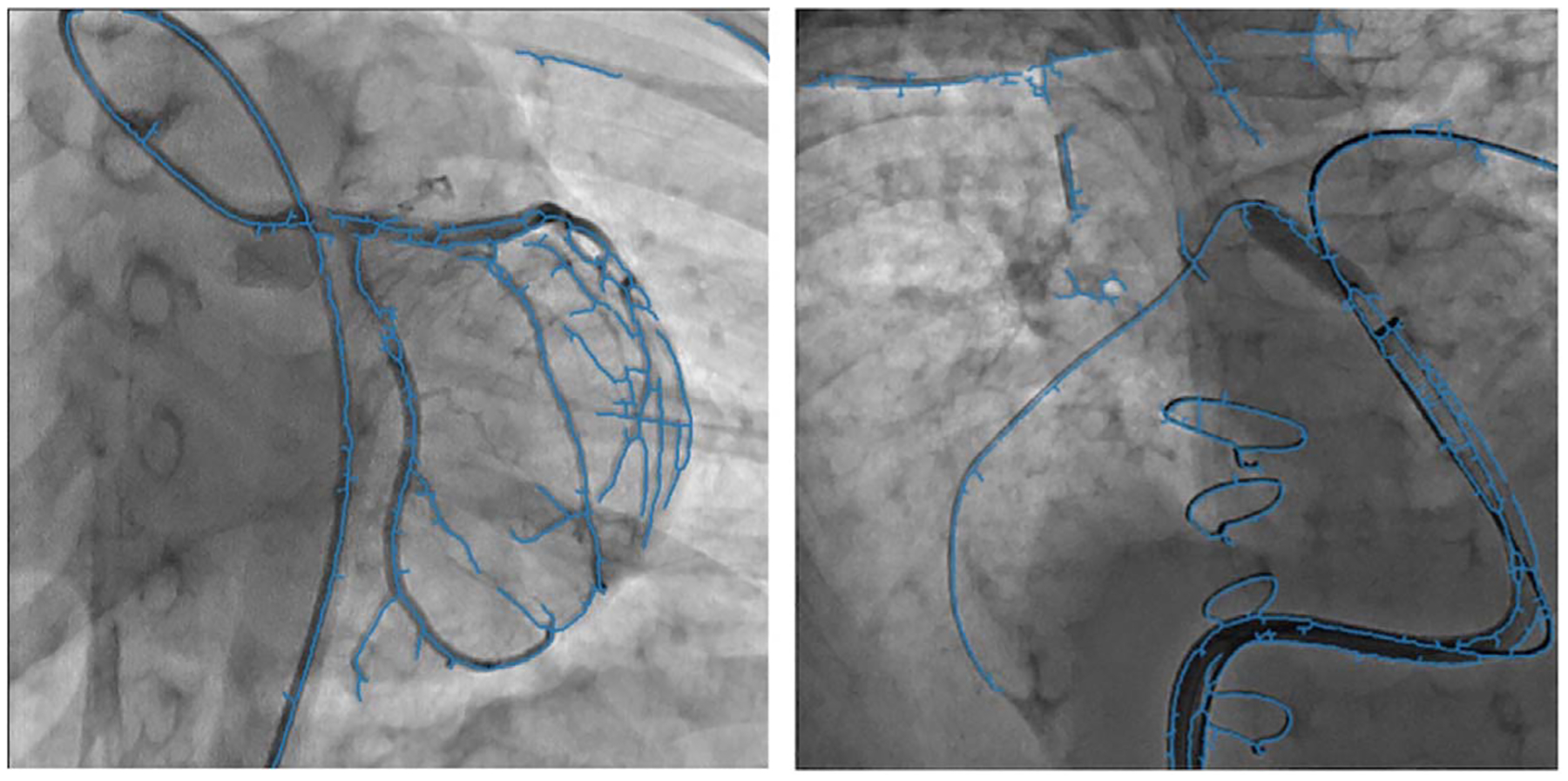
Two different patient data samples for overlaying the transformed vessels with predicted transformation parameters (blue colored vessel) with the original transformed images.

We applied the predicted parameters in matrix form to transform the arteries’ centerlines and overlaid the transformed centerlines with predicted parameters on the distance transform of the extracted centerlines from the original images. Figures 12, 13 and 14 show the overlay of the transformed segmented vessels with predicted transformation parameters and the original transformed images for simulated and patient data, respectively.

Therefore, we first evaluated our results by computing the Mean Absolute Error between the predicted TP values and the ground truth TP (resulting from CPD registration) (tables 1 and 2), and then we compared our results to the original images by applying for the CPD registration on the originally extracted and predicted centerlines (3). Apart from the simulated data, we validated our results by applying the method on 10 real patients, with a more realistic and noisy segmentation of the moving objects.

As shown in the results presented in table 3, we obtained a low accumulated error for the prediction of the transformation matrix using the distance transform of the original segmented arteries for both simulated data with different motions as well as the patient data samples.

## 5. Discussion

We have presented a learning-based patient-specific cardio-respiratory motion prediction method using a simple LSTM model. This model can predict the temporal and spatial changes for moving objects (contrasted arteries and/or medical devices) in a sequence. This approach is an extension of our previous work (Azizmohammadi *et al* 2019). We applied the same pre-processing and motion feature extraction while we extended the data for development and validation. Here are the differences between the newly presented work and our previous study: (1) The preliminary results of the first model were obtained by developing and validating the deep LSTM model on only 3 synthetic datasets (only adult patients). The new results were obtained on 64 synthetic datasets (pediatric and adults), including adults and pediatric patients. Then, we validated our final results using actual patient data (10 different sequences from different patients). Hence, the proposed motion prediction method was validated on a wide range of simulated cardiac and respiratory rates for pediatric and adult phantoms as well as a patient dataset. The resulting prediction error of this method (average 0.58 mm) on the patient data is promising since, in a few samples for the patient dataset, we had an unexpected patient motion that appeared as irregularity in the cardio-respiratory motion signal. Figure 11 represents the 2D translation of the moving objects in the sequences, there are irregularities in the cardio-respiratory motion signal extracted from a 150-frame sequence. Nevertheless, the imposed patient movement did not affect the predictions. (2) The preliminary deep LSTM model was a many-to-one structure. The input is a sequence of transformation matrices, and the output is the prediction of the single matrix at position *n* + 1. In a many-to-one model, to generate the output, the final input must be entered into the model. The performance of the updated model was improved by extending it to have multiple output predictions using a many-to-many structure. The many-to-many model generates the output whenever each input is read. Thus, a many-to-many model can understand the feature of each token (matrix) in the input sequence. Compared to other learning-based predictive methods with a 2mm prediction error (Lee and Motai 2014), our both models have achieved a prediction error of less than 1 mm (0.58 mm-new approach).

For the patient data, the medical devices such as guide wires or catheters were considered as moving objects in a sequence since they are continuously visible and contrasted under the X-ray images. Hence, the visible devices can represent the movement of arteries, while the contrasted arteries gradually become faded through a sequence as they lose their contrast. Motion prediction is thus not completely dependent on the visibility of the contrasted arteries with the contrast agent.

The presented motion tracking approach can be applied for the estimation of future heart trajectory in robotic-assisted operations on a free-beating heart as a robust prediction algorithm. The training time for each sequence depends on the length of the input sequence. We trained the model using CPU, and each step for training took 6ms. Aside from other factors such as the hardware (CPU or GPU) and optimized implementation, the time complexity for training would grow linearly by increasing the length of the input sequence. Moreover, no additional imaging modality and preoperative information are required for motion tracking using this approach. Not only this model-free cardio-respiratory motion prediction approach can facilitate the navigation process of cardiac interventions but also potentially aids in reducing the required amount of contrast agent and radiation dose for cardiac interventions.

The accuracy of the prediction indirectly depends on the accuracy of the segmentation and registration algorithms in the pre-processing steps. Segmentation and centerline extraction for the moving objects (arteries and medical devices) was challenging, especially for the patient data. In our preprocessing steps, we used the fixed parameters for simulated and patient datasets. With the connected component approach, small objects were removed, and the centerline of the segmented objects was extracted using skeletonization. Hence, the segmentation and centerline extraction error accumulated into the final prediction error. In figure 14, the extracted centerlines for the arteries are the edges of the contrasted guide wires and not the actual centerlines of the arteries. Future work will include investigating an accurate state-of-the-art method based on a Convolutional Neural Network (CNN) for automatizing the segmentation in our pre-processing steps. Moreover, we are planning to integrate this motion prediction approach into an End-to-End system for xXray image prediction.

## 6. Conclusion

This study investigated an RNN-based motion prediction model that can be used for cardio-respiratory motion tracking in the navigation of cardiac interventions with X-ray angiographies. We developed a simple LSTM model that can predict the 2D transformation of visible moving objects in an X-ray sequence. This patient-specific motion prediction approach can estimate the cardio-respiratory motion signal with a less than 1 mm prediction error.

## Figures and Tables

**Table 1.** MAE between the ground truth and predicted transformation parameter values for simulated data.

Both motions						
MAE (pixel)	Tx	Ty	a00	a01	a10	a11
Mean	0.24	0.21	0.32	0.35	0.32	0.32
Max	0.41	0.42	0.40	0.51	0.52	0.51
Min	0.00	0.00	0.06	0.07	0.02	0.06
Only cardiac motion						
Mean	0.13	0.11	0.14	0.12	0.13	0.13
Max	0.35	0.43	0.42	0.42	0.44	0.45
Min	0.00	0.00	0.00	0.06	0.00	0.05
Only Respiratory motion						
Mean	0.10	0.13	0.11	0.14	0.11	0.15
Max	0.21	0.43	0.37	0.50	0.41	0.45
Min	0.00	0.00	0.00	0.00	0.00	0.00

**Table 2.** MAE between the ground truth and predicted of transformation parameter values for patient data over 10 examples.

Patient data						
MAE (pixel)	Tx	Ty	a00	a01	a10	a11
Mean	0.19	0.20	0.20	0.20	0.18	0.17
Max	0.55	0.45	0.54	0.55	0.39	0.47
Min	0.02	0.03	0.01	0.00	0.02	0.01

**Table 3.** Average overall sample distance transform error of the original centerline image to the predicted transformed one in mm.

Mean DT error(mm)	Both	Cardiac	Respiratory	Patient data (Both)
	0.39mm	0.33 mm	0.47 mm	0.58 mm

## References

[R1] Ackermann MA and Ender JK 2019 Recent developments in catheter-based cardiac procedures Anesthesiology Clin. 37 621–38 10.1016/j.anclin.2019.08.014 31677682

[R2] Ambrosini P, Ruijters D, Niessen WJ, Moelker A and Walsum TV 2017 Fully automatic and real-time catheter segmentation in X-ray fluoroscopy Int. Conf. on Medical Image Computing and Computer-Assisted Intervention Springer pp 577–85 (https://springerprofessional.de/en/fully-automatic-and-real-time-catheter-segmentation-in-x-ray-flu/14979058)

[R3] Azizmohammadi F, Martin R, Miro J and Duong L 2019 Model-free cardiorespiratory motion prediction from X-ray angiography sequence with lstm network 2019 XLI Annual Int. Conf. of the IEEE Engineering in Medicine and Biology Society (EMBC) IEEE pp 7014–8 10.1109/EMBC.2019.8857798 31947453

[R4] Baka N, Lelieveldt B, Schultz C, Niessen W and Van Walsum T 2015 Respiratory motion estimation in X-ray angiography for improved guidance during coronary interventions Phys. Med. Biol 60 36179 10.1088/0031-9155/60/9/3617 25860615

[R5] Dauer LT 2011 Radiation dose management for fluoroscopically-guided interventional procedures (10.1097/HP.0b013e3182289c31)

[R6] Dieterich S and Murphy MJ 2006 Comparative performance of linear and nonlinear neural networks to predict irregular breathing Phys. Med. Biol 51 5903–14 17068372 10.1088/0031-9155/51/22/012

[R7] Ernst F and Schweikard A 2009 Forecasting respiratory motion with accurate online support vector regression (svrpred) Int. J. Comput. Assist. Radiol. Surg 4 439–47 20033526 10.1007/s11548-009-0355-5

[R8] Feldkamp LA and Puskorius GV 1994 IEEE Trans. Neural Netw. 5, 279297 (1994) 5 279–97 10.1109/72.279191 18267797

[R9] Gierga DP, Brewer J, Sharp GC, Betke M, Willett CG and Chen GT 2005 The correlation between internal and external markers for abdominal tumors: implications for respiratory gating Int. J. Radiat. Oncol.* Biol.* Phys 61 1551–8 15817361 10.1016/j.ijrobp.2004.12.013

[R10] Jung BH, Kim BH and Hong SM 2013 Respiratory motion prediction with extended kalman filters based on local circular motion model Int. J. Bio-Sci. Bio-Technol 5 51–8

[R11] Kalet A, Sandison G, Wu H and Schmitz R 2010 A state-based probabilistic model for tumor respiratory motion prediction Phys. Med. Biol 55 7615 21113094 10.1088/0031-9155/55/24/015

[R12] Kesner SB and Howe RD 2010 Design and control of motion compensation cardiac catheters 2010 IEEE Int. Conf. on Robotics and Automation IEEE pp 1059–65 10.1109/ROBOT.2010.5509250 PMC5785916 29375926

[R13] Lee SJ and Motai Y 2014 Prediction of respiratory motion Prediction and Classification of Respiratory Motion (Berlin: Springer) pp 7–37

[R14] Ma Y, King AP, Gogin N, Gijsbers G, Rinaldi CA, Gill J, Razavi R and Rhode KS 2011 Clinical evaluation of respiratory motion compensation for anatomical roadmap guided cardiac electrophysiology procedures IEEE Trans. Biomed. Eng 59 122–31 21926014 10.1109/TBME.2011.2168393

[R15] McClelland JR, Hawkes DJ, Schaeffter T and King AP 2013 Respiratory motion models: a review Med. Image Anal 17 19–42 23123330 10.1016/j.media.2012.09.005

[R16] Murphy D and Murphy MJ 2009 Optimization of an adaptive neural network to predict breathing Phys. Med. Biol 40–7 10.1118/1.3026608 PMC2739312 19235372

[R17] Myronenko A and Song X 2010 Point set registration: Coherent point drift IEEE Trans. Pattern Anal. Mach. Intell 32 2262–75 20975122 10.1109/TPAMI.2010.46

[R18] Nehrke K, Bornert P, Manke D and Bock JC 2001 Free-breathing cardiac mr imaging: study of implications of respiratory motioninitial results Radiology 220 810–5 11526286 10.1148/radiol.2203010132

[R19] Oshinski JN, Hofland L, Mukundan S Jr., Dixon WT, Parks WJ and Pettigrew RI 1996 Two-dimensional coronary mr angiography without breath holding Radiology 201 737–43 8939224 10.1148/radiology.201.3.8939224

[R20] Respiratory motion prediction by using the adaptive neuro fuzzy inference system (ANFIS) 2005 Respiratory motion prediction by using the adaptive neuro fuzzy inference system (anfis) Phys. Med. Biol 50 4721 16177500 10.1088/0031-9155/50/19/020

[R21] Riaz N, Shanker P, Wiersma R, Gudmundsson O, Mao W, Widrow B and Xing L 2009 Predicting respiratory tumor motion with multidimensional adaptive filters and support vector regression Phys. Med. Biol 54 5735 19729711 10.1088/0031-9155/54/19/005 PMC12165777

[R22] Roujol S, Anter E, Josephson ME and Nezafat R 2013 Characterization of respiratory and cardiac motion from electro-anatomical mapping data for improved fusion of mri to left ventricular electrograms PLoS One 8 e78852 24250815 10.1371/journal.pone.0078852 PMC3826750

[R23] Ruan D and Keall P 2010 Online prediction of respiratory motion: multidimensional processing with low-dimensional feature learning Phys. Med. Biol 55 3011 20442460 10.1088/0031-9155/55/11/002 PMC2975024

[R24] Schilling K, Ma L and Herrmann C 2007 Modeling and prediction of lung tumor motion for robotic assisted radiotherapy IEEE/RSJ Int. Conf. on Intelligent Robots and Systems pp 189–94

[R25] Schneider M, Sundar H, Liao R, Hornegger J and Xu C 2010 Model-based respiratory motion compensation for image-guided cardiac interventions 2010 IEEE Computer Society Conf. on Computer Vision and Pattern Recognition IEEE pp 2948–54

[R26] Schweikard A, Glosser G, Bodduluri M, Murphy MJ and Adler JR 2000 Robotic motion compensation for respiratory movement during radiosurgery Comput. Aided Sur 5 263–77 10.1002/1097-0150(2000)5:4<263::AID-IGS5>3.0.CO;2-2 11029159

[R27] Scott LB, Gravely S, Sexton TR, Brzostek S and Brown DL 2013 Examining the effect of a patient navigation intervention on outpatient cardiac rehabilitation awareness and enrollment J. Cardiopulmonary Rehabil. Prevention 33 281 10.1097/HCR.0b013e3182972dd6 PMC3759655 23823904

[R28] Segars W, Sturgeon G, Mendonca S, Grimes J and Tsui BM 2010 4D XCAT phantom for multimodality imaging research Med. Phys 37 4902–15 20964209 10.1118/1.3480985 PMC2941518

[R29] Segars W 2013 Population of anatomically variable 4D XCAT adult phantoms for imaging research and optimization Med. Phys 40 043701 23556927 10.1118/1.4794178 PMC3612121

[R30] Sharp GC, Jiang SB, Shimizu S and Shirato H 2004 Prediction of respiratory tumour motion for real-time image-guided radiotherapy Phys. Med. Biol 49 425 15012011 10.1088/0031-9155/49/3/006

[R31] Shechter G, Ozturk C, Resar JR and McVeigh ER 2004 Respiratory motion of the heart from free breathing coronary angiograms IEEE Trans. Med. Imaging 23 1046–56 15338737 10.1109/TMI.2004.828676 PMC2494710

[R32] Shechter G, Shechter B, Resar JR and Beyar R 2005 Prospective motion correction of X-ray images for coronary interventions IEEE Trans. Med. Imaging 24 441–50 15822802 10.1109/tmi.2004.839679

[R33] Taylor AM, Jhooti P, Wiesmann F, Keegan J, Firmin DN and Pennell DJ 1997 Mr navigator-echo monitoring of temporal changes in diaphragm position: implications for mr coronary angiography J. Magn. Reson. Imaging 7 629–36 9243380 10.1002/jmri.1880070404

[R34] Timinger H, Krueger S, Dietmayer K and Borgert J 2005 Motion compensated coronary interventional navigation by means of diaphragm tracking and elastic motion models Phys. Med. Biol 50 491 15773725 10.1088/0031-9155/50/3/007

[R35] Werner R, Ehrhardt J, Schmidt R and Handels H 2009 Patient-specific finite element modeling of respiratory lung motion using 4d ct image data Med. Phys 36 1500–11 19544766 10.1118/1.3101820

